# Correction to: The role of microglia membrane potentialin chemotaxis

**DOI:** 10.1186/s12974-021-02093-3

**Published:** 2021-01-28

**Authors:** Laura Laprell, Christian Schulze, Marie-Luise Brehme, Thomas G. Oertner

**Affiliations:** Institute for Synaptic Physiology, Center for Molecular NeurobiologyHamburg (ZMNH), University Medical Center Hamburg-Eppendorf, Falkenried94, 20251 Hamburg, Germany

**Correction to: J Neuroinflammation 18, 21 (2021)**

**https://doi.org/10.1186/s12974-020-02048-0**

Following publication of the original article [1], the authors noticed an incorrect Fig. 1 image and incorrect panel “e” background color on the image of Fig. 3 in the published version of this article. Presented here are the corrected Figs. 1 and 3. The original article has been updated.

**Fig. 1:**
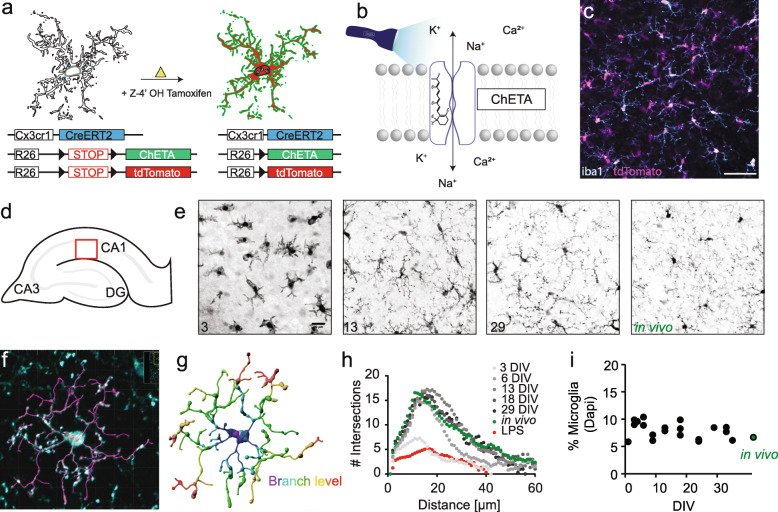
Microglia-specific ChETA expression in organotypic hippocampal slice cultures. **a** Schematic overview of microglia-specific expression. The microglia-driver line Cx3cr1-CreERT2 (blue) is crossed with a reporter mouse line (R26-LSL-tdTomato, red) and the ChETA mouse line (R26-LSL-ChETA, green). After injection of (Z)-4-hydroxytamoxifen, the tdTomato and ChETA are expressed in microglia. **b** Illustration of the Channelrhodopsin-variant ChETA activated by blue light. Scale bar 25 μm. **c** Immunostaining using antibodies against the reporter (tdTomato - red) and microglia (iba1 - cyan). **d** Graphic illustration of the hippocampal structure and the investigated area for microglia morphology in e (red square). **e** Z-projection of confocal images acquired for Sholl analysis of microglia at 3, 13, 29 DIV, and in vivo. Scale bar: 25 μm. **f** Confocal image of a microglia cell in organotypic slice culture which was fixed with PFA and stained against the microglia marker iba1. Overlay with IMARIS analysis (magenta). **g** Result of microglia branch detection with color coding by branching level. **h** Sholl analysis of microglia over time (number of intersections versus distance from cell body). **i** Quantification of % microglia cells between dentate gyrus and CA1 relative to total cell count (DAPI)

**Fig. 3:**
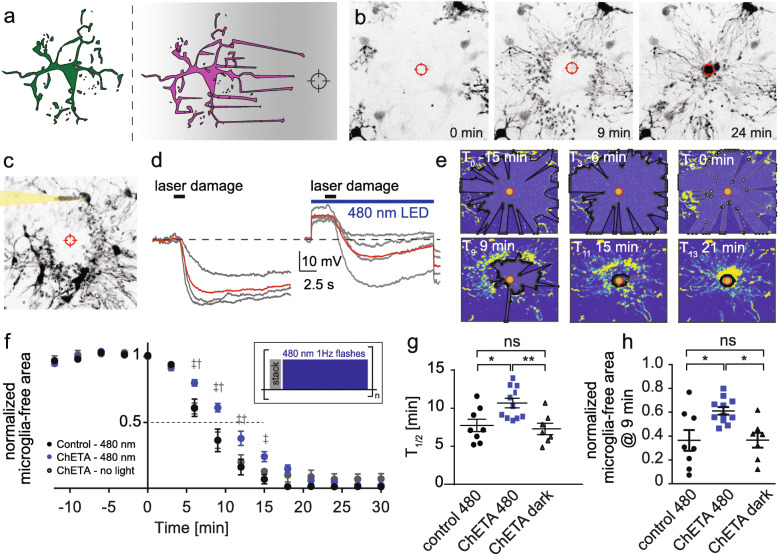
Optogenetic microglia depolarization decelerates chemotactic response kinetics. **a** Graphic illustration and representative images of microglia chemotaxis towards an induced laser-damage. **b** Two-photon maximum projections of the chemotactic response 0, 9, and 24 min after the laser-damage. **c** Two-photon z-projection of a patched microglia during chemotaxis. **d** Voltage-clamp recordings of patched microglia during chemotaxis. Gray: Individual microglial responses from four experiments, red: Average of all experiments. Left: no light stimulation during laser-damage, right: with light stimulation during laser damage. **e** Automated MATLAB analysis of chemotaxis quantified as the reduction in microglia-free area around the laser damage (black polygon) at different time points of the experiment. **f** Relative laser damage response measured as microglia-free area. Black: Control slices (no construct) with light stimulation (n = 8 areas, 5 slices). Gray: Experiments with ChETA expression in microglia, but without light stimulation (n = 7 areas, 4 slices). Blue: Slices with ChETA expression in microglia combined light stimulation (n = 11 areas, 7 slices). Insert: Graphic representation of light stimulation protocol between stack acquisitions. 2-way ANOVA (‡ control480 – ChETA480, p < 0.001, († ChETA no light – ChETA480, p < 0.01). **g** Time to 50% engulfment was prolonged by optogenetic depolarization. **h** 9 min after injury, the microglia-free area was larger when microglia were depolarized. **g, h** One-way ANOVA with Tukey’s post hoc comparison (*p < 0.05, **p < 0.01, ***p < 0.001)
