# Supplementary material for: The role of microglia membrane potential in chemotaxis
Source: J Neuroinflammation. 2021 Jan 10;18:21. doi: 10.1186/s12974-020-02048-0 (PMC7798195; doi:10.1186/s12974-020-02048-0)
Supplement: Supplementary file 1 — Additional file 1 Cell parameters in microglia of male and female mice show no differences in slice cultures. Duration of slice culture in vitro has no significant effect on microglia membrane potential. (a) Membrane resistance (n = 14/9, male/female), (b) Membrane capacitance (n = 9/12, male/female), (c) Membrane resting potential (n = 12/8, male/female), (d) Membrane resting potential vs. days in vitro (n = 23). Spearman’s ρ = -0.24, P = 0.27, n = 23 experiments. [file 12974_2020_2048_MOESM1_ESM.docx]

# Supplementary Information


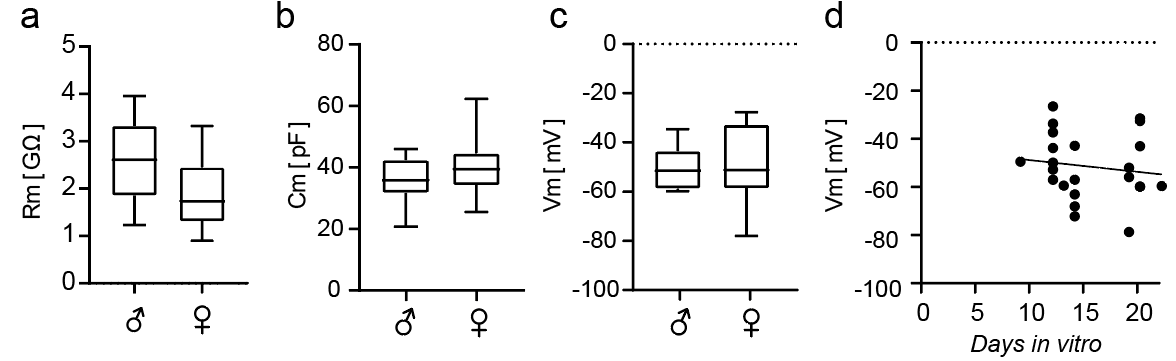


**Additional file 1.** Cell parameters in microglia of male and female mice show no differences in slice cultures. Duration of slice culture in vitro has no significant effect on microglia membrane potential. (a) Membrane resistance (n=14/9, male/female), (b) Membrane capacitance (n=9/12, male/female), (c) Membrane resting potential (n=12/8, male/female), (d) Membrane resting potential vs. days in vitro (n=23). Spearman’s ρ = -0.24, P = 0.27, n = 23 experiments.
