# Supplementary material for: The role of microglia membrane potential in chemotaxis
Source: J Neuroinflammation. 2021 Jan 10;18:21. doi: 10.1186/s12974-020-02048-0 (PMC7798195; doi:10.1186/s12974-020-02048-0)
Supplement: Supplementary file 2 — Additional file 2 Optogenetic depolarization mildly affects microglia baseline properties. (A) Superimposed images of the same microglia at two timepoints one minute apart showing process movement during surveillance (purple, retracted; blue, extended and gray stable processes). (B-C) Relative microglia ramification (B) and surveillance index (C) over the time course of 50 min (ChETA_480, n = 4 microglia in 3 slices, control_480, n = 8 microglia in 5 slices). Light application (20 min with 480 nm) was started after 10 min of baseline recording. Statistical analysis: Two-way ANOVA with Bonferroni post-hoc comparison (*p < 0.05) (D) Overview table of relative ramification and surveillance indices in the 10 min before light application and the first 10 minutes of light application (Average ± SEM). [file 12974_2020_2048_MOESM2_ESM.docx]

**
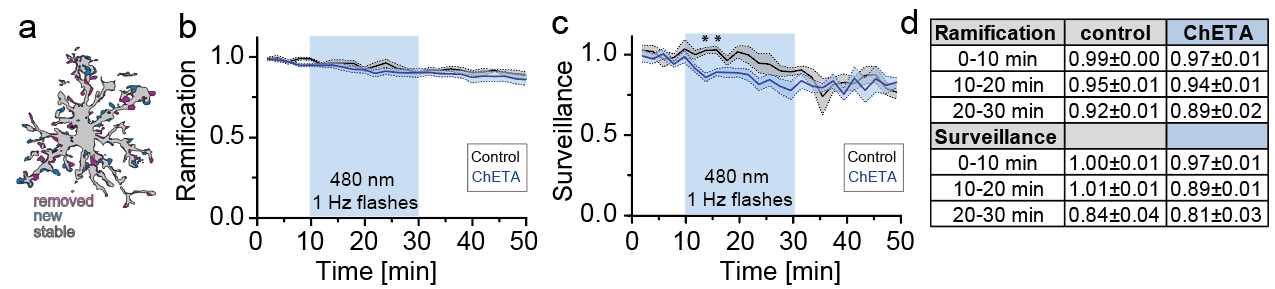
Additional file 2 – Optogenetic depolarization mildly affects microglia baseline properties**. (A) Superimposed images of the same microglia at two timepoints one minute apart showing process movement during surveillance (purple, retracted; blue, extended and gray stable processes). (B-C) Relative microglia ramification (B) and surveillance index (C) over the time course of 50 min (ChETA_480, n = 4 microglia in 3 slices, control_480, n = 8 microglia in 5 slices). Light application (20 min with 480 nm) was started after 10 min of baseline recording. Statistical analysis: Two-way ANOVA with Bonferroni post-hoc comparison (*p < 0.05) (D) Overview table of relative ramification and surveillance indices in the 10 min before light application and the first 10 minutes of light application (Average ± SEM).
