# Supplementary material for: The role of microglia membrane potential in chemotaxis
Source: J Neuroinflammation. 2021 Jan 10;18:21. doi: 10.1186/s12974-020-02048-0 (PMC7798195; doi:10.1186/s12974-020-02048-0)
Supplement: Supplementary file 3 — Additional file 3 Microglia response time is independent from distance of ablation, temperature, sex, or days in vitro. (a) Amount of hyperpolarization of microglia membrane potential induced by laser-damage with and without activation of ChETA (n = 5 controls, n = 4 ChETA, DIV 20-22) (b) T1/2 plotted against the distance of microglia processes from the laser damage (Spearman’s ρ = 0.15, P = 0.47, n = 26 experiments). (c) T1/2 plotted against the temperature of the extracellular solution (Spearman’s ρ =−0.24, P = 0.25, n = 26 experiments). (d) T1/2 plotted for microglia in slice cultures from male (n = 5/4 control/light) and female (n = 8/7 control/light) animals. (e) T1/2 plotted against DIV for control slices (black) and ChETA slices (blue). Control: Spearman’s ρ = -0.2, P = 0.66, n = 8 experiments, ChETA: Spearman’s ρ = -0.6, P = 0.06, n = 11 experiments. [file 12974_2020_2048_MOESM3_ESM.docx]

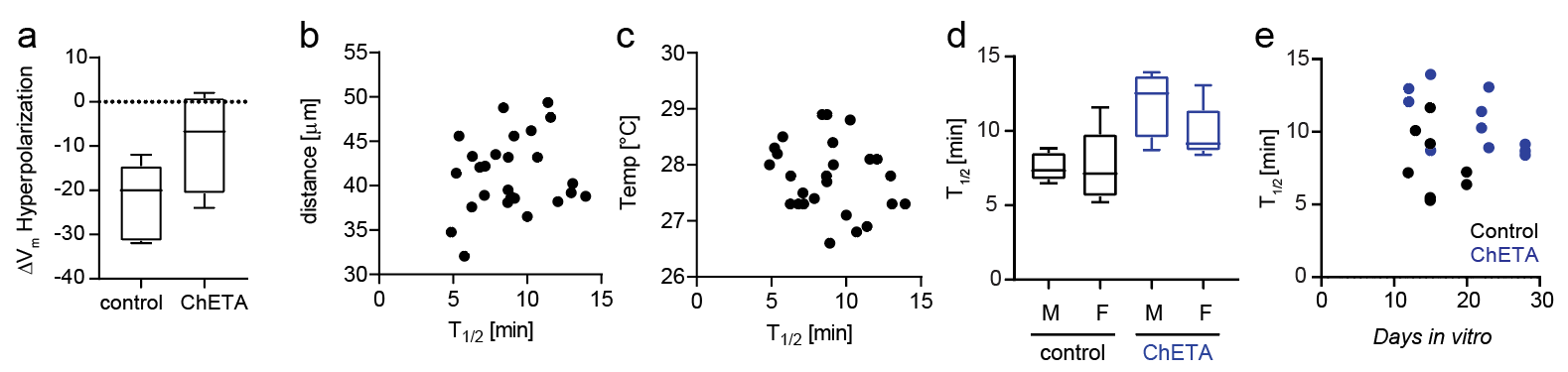


**Additional file 3 - Microglia response time is independent from distance of ablation, temperature, sex, or days *in vitro*.** (a) Amount of hyperpolarization of microglia membrane potential induced by laser-damage with and without activation of ChETA (n=5 controls, n=4 ChETA, DIV 20-22) **(**b) T_1/2_ plotted against the distance of microglia processes from the laser damage (Spearman’s ρ = 0.15, P = 0.47, n = 26 experiments). (c) T_1/2_ plotted against the temperature of the extracellular solution (Spearman’s ρ =$-$0.24, P = 0.25, n = 26 experiments). (d) T_1/2_ plotted for microglia in slice cultures from male (n=5/4 control/light) and female (n=8/7 control/light) animals. (e) T_1/2_ plotted against DIV for control slices (black) and ChETA slices (blue). Control: Spearman’s ρ = -0.2, P = 0.66, n = 8 experiments, ChETA: Spearman’s ρ = -0.6, P = 0.06, n = 11 experiments.
