# Supplementary material for: The role of microglia membrane potential in chemotaxis
Source: J Neuroinflammation. 2021 Jan 10;18:21. doi: 10.1186/s12974-020-02048-0 (PMC7798195; doi:10.1186/s12974-020-02048-0)
Supplement: Supplementary file 4 — Additional file 4. ArchT effectively hyperpolarizes microglia under resting conditions. (a) Immunohistochemical staining of ArchT (green) in microglia (red). (b) Light -induced currents in microglia expressing ArchT. Red lines: Average time course. (c) Light-dose dependence of currents induced in microglia. (d) Prolonged light stimulation induces robust, lasting currents in microglia. (e-f) Repetitive light stimulation induces stable light-induced currents in microglia. [file 12974_2020_2048_MOESM4_ESM.docx]

**
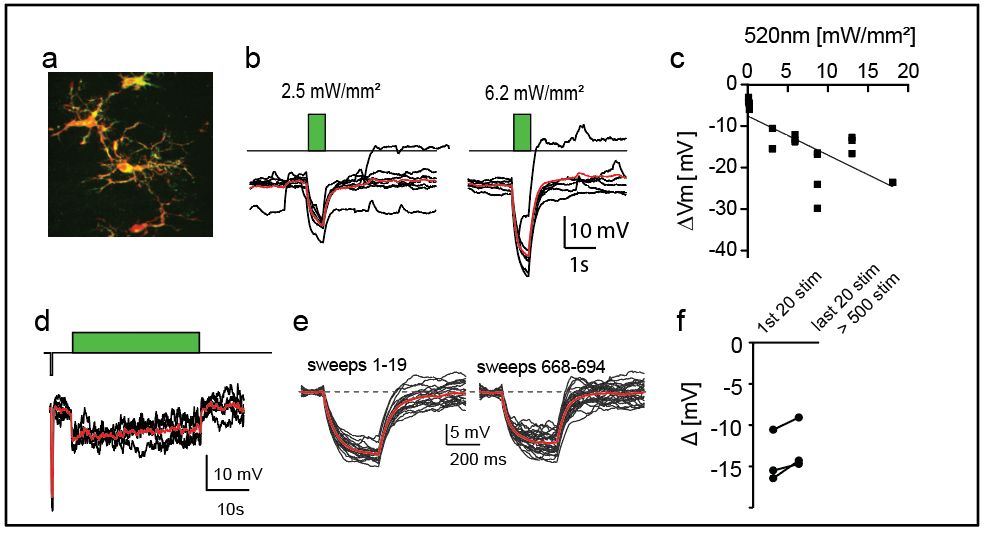
**

**Additional file 4 - ArchT effectively hyperpolarizes microglia under resting conditions**. (a) Immunohistochemical staining of ArchT (green) in microglia (red). (b) Light -induced currents in microglia expressing ArchT. Red lines: Average time course. (c) Light-dose dependence of currents induced in microglia. (d) Prolonged light stimulation induces robust, lasting currents in microglia. (e-f) Repetitive light stimulation induces stable light-induced currents in microglia.
