# Supplementary material for: The role of microglia membrane potential in chemotaxis
Source: J Neuroinflammation. 2021 Jan 10;18:21. doi: 10.1186/s12974-020-02048-0 (PMC7798195; doi:10.1186/s12974-020-02048-0)
Supplement: Supplementary file 5 — Additional file 5 ArchT activation during laser damage does not further increase hyperpolarization and has no effect on laser-damage induced chemotactic responses. (a) Schematic drawing of a chemotactic microglia response. (b) ATP-mediated hyperpolarization in microglia. Red line: Average time course. (c) ArchT light- induced hyperpolarization. (d) Combination of ArchT induced hyperpolarization with laser-damage response. (e) Chemotactic response over time while constantly applying light during acquisition of stacks. (n = 9 (5 female, 4 male)) (f) T1/2 of microglia responses in control and ArchT expressing cultures. [file 12974_2020_2048_MOESM5_ESM.docx]

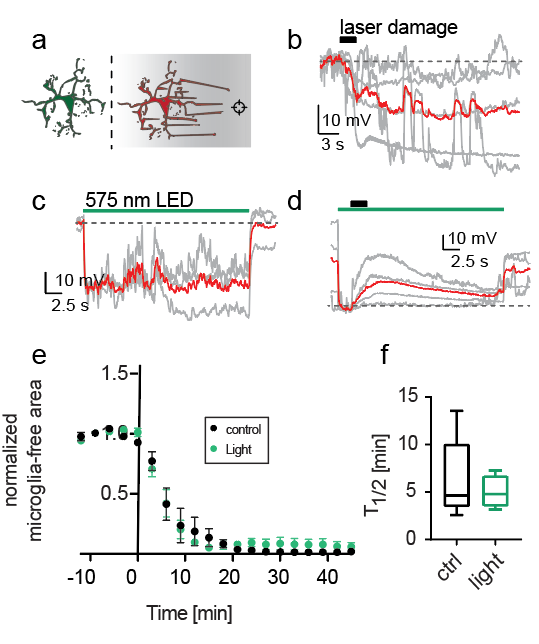


**Additional file 5 - ArchT activation during laser damage does not further increase hyperpolarization and has no effect on laser-damage induced chemotactic responses.** (a) Schematic drawing of a chemotactic microglia response. (b) ATP-mediated hyperpolarization in microglia. Red line: Average time course. (c) ArchT light- induced hyperpolarization. (d) Combination of ArchT induced hyperpolarization with laser-damage response. (e) Chemotactic response over time while constantly applying light during acquisition of stacks. (n = 9 (5 female, 4 male)) (f) T1/2 of microglia responses in control and ArchT expressing cultures.
