# Supplementary material for: The role of microglia membrane potential in chemotaxis
Source: J Neuroinflammation. 2021 Jan 10;18:21. doi: 10.1186/s12974-020-02048-0 (PMC7798195; doi:10.1186/s12974-020-02048-0)
Supplement: Supplementary file 6 — Additional file 6 Microglia response kinetics towards tissue damage are slowed down in nominally Ca2+ free extracellular solution. (a) Relative laser damage response measured as microglia-free area in HEPES solution with 2 mM Ca2+ (n = 8, 3 slices, female, DIV 15-21) (black) and in nominally Ca2+-free HEPES solution (n = 8, 3 slices, female, DIV 15-16) (red). 2-way ANOVA (** p < 0.01, *** p < 0.001). (b-c) Summary of T1/2 and microglia-free area slope (0-7 min). One-way ANOVA with Tukey’s post-hoc comparison (* p < 0.05, *** p < 0.001). (d) Z-projection of individual areas 7 min after laser damage. Black: microglia processes extension in control conditions with 2 mM Ca2+. Red: microglia process extension under nominally Ca2+-free conditions. [file 12974_2020_2048_MOESM6_ESM.docx]

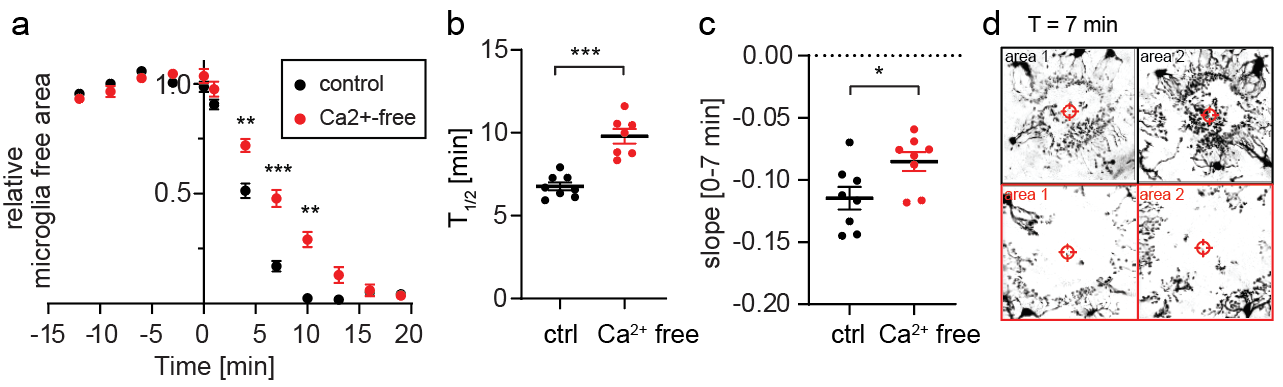


**Additional file 6 - Microglia response kinetics towards tissue damage are slowed down in nominally Ca^2+^ free extracellular solution.** (a) Relative laser damage response measured as microglia-free area in HEPES solution with 2 mM Ca^2+^ (n = 8, 3 slices, female, DIV 15-21) (black) and in nominally Ca^2+^-free HEPES solution (n = 8, 3 slices, female, DIV 15-16) (red). 2-way ANOVA (** p < 0.01, *** p < 0.001). (b-c) Summary of T_1/2_ and microglia-free area slope (0-7 min). One-way ANOVA with Tukey’s post-hoc comparison (* p < 0.05, *** p < 0.001). (d) Z-projection of individual areas 7 min after laser damage. Black: microglia processes extension in control conditions with 2 mM Ca^2+^. Red: microglia process extension under nominally Ca^2+^-free conditions.
